# *ERCC1* polymorphism and its expression associated with ischemic stroke in Chinese population

**DOI:** 10.3389/fneur.2022.998428

**Published:** 2023-01-12

**Authors:** Xiao-Dong Deng, Jian-Lin Ke, Tai-Yu Chen, Qin Gao, Zhuo-Lin Zhao, Wei Zhang, Huan Liu, Ming-Liang Xiang, Li-Zhen Wang, Ying Ma, Yun Liu

**Affiliations:** ^1^Department of Forensic Pathology, School of Basic Medical Science and Forensic Medicine, North Sichuan Medical College, Nanchong, China; ^2^Department of Intergrated Western and Chinese Colorectal and Anal Surgery, Affiliated Hospital of North Sichuan Medical College, Nanchong, China; ^3^Department of Internal Medicine, Nanchong Jialing District People's Hospital, Nanchong, China; ^4^Department of Neurology, The Second People's Hospital of Yibin, Yibin, China; ^5^Department of Preventive Medicine, North Sichuan Medical College, Nanchong, China; ^6^Department of Neurology, Affiliated Hospital of North Sichuan Medical College, Nanchong, China; ^7^Sichuan Key Laboratory of Medical Imaging, Affiliated Hospital of North Sichuan Medical College, Nanchong, China

**Keywords:** ischemic stroke, polymorphism, susceptibility, expression, *ERCC1*

## Abstract

**Background:**

Excision repair cross-complementing group 1 (*ERCC1*) was considered a potential candidate gene for ischemic stroke, and its polymorphisms might be associated with the susceptibility to ischemic stroke.

**Methods:**

A total of 513 patients with ischemic stroke and 550 control subjects were recruited. The expression levels of *ERCC1* messenger RNA (mRNA) in peripheral blood mononuclear cells and its protein in plasma were detected by quantitative real-time PCR (*qPCR*) and enzyme-linked immunosorbent assay (*ELISA*), respectively. *Rs3212986* polymorphism of *ERCC1* was detected by PCR-restriction fragment length polymorphism (*RFLP-PCR*) and was confirmed by sequencing. The association between the *ERCC1 rs3212986* polymorphism or its expression and ischemic stroke was further analyzed.

**Results:**

The *ERCC1* mRNA level in patients with ischemic stroke was lower than that in the control group (*P* < 0.05). However, the *ERCC1* protein level in patients with ischemic stroke was higher than that in the control group (*P* < 0.05). The A allele of *rs3212986* was associated with increased ischemic stroke risk (OR = 1.287, 95% CI = 1.076–1.540, *P* = 0.006). The association between *rs3212986* polymorphism and ischemic stroke susceptibility was found in both recessive (OR = 2.638, 95% CI = 1.744–3.989, *P* < 0.001) and additive models (OR = 1.309, 95% CI = 1.028–1.667, *P* = 0.031), respectively. Similar results were obtained in the recessive model (OR = 2.015, 95% CI = 1.087–3.704, *P* = 0.026) after adjusting for demographic information and other variables. Additionally, the level of *ERCC1* mRNA in the CC/CA genotype was higher than that in the AA genotype (*P* < 0.05).

**Conclusion:**

It was suggested that the *ERCC1 rs3212986* polymorphism was associated with ischemic stroke susceptibility in a Chinese Han population and that an A allele of *rs3212986* was related to increased ischemic stroke risk. The altered *ERCC1* expression level caused by the *rs3212986* polymorphism might participate in the pathophysiological process of ischemic stroke.

## 1. Introduction

Ischemic stroke is a common age-related cerebrovascular disorder, accounting for 87% of all strokes, with a high disability and mortality rate, causing a huge burden to the economy and society (1, 2). However, the pathophysiological mechanism of ischemic stroke remains unclear (3). Recently, genomic instability due to the unresolved accumulation of DNA variants was considered one of the contributors to age-related diseases (4). Vascular and endothelial function deteriorated with age because those unresolved DNA variants gradually increased, and was hypothesized as a key risk factor in the development and progression of age-related diseases, such as cardiovascular and cerebrovascular diseases. The unresolved accumulations of DNA variants promoted endothelial cell dysfunction, vascular senescence, and plaque rupture and eventually contributed to heart attacks or ischemic strokes (5). Durik et al. conducted a study to investigate whether DNA damage plays an important role in age-related vascular dysfunction in the nucleotide excision repair (NER)-defect mouse model *via* inactivated excision repair cross-complementing group 1 (*ERCC1*), a key component as a highly conserved rate-limiting enzyme in NER, and found that *ERCC1*^*d*/−^ mice accelerated the development of vasodilator dysfunction and increased vascular senescence and stiffness. In addition, to discuss if DNA damage-related gene variants could have an impact on human vascular disease and in line with our murine phenotype, they performed genetic studies to evaluate the association of NER-related gene variations with carotid-femoral pulse wave velocity and found a significant association of a SNP (*rs2029298*) of the damage-specific DNA binding protein 2 (*DDB2*) gene required for DNA binding in NER with carotid-femoral pulse wave velocity. These results suggested that genomic instability due to NER-related gene variations played a key role in age-dependent vascular dysfunction as observed in animal models and in humans (6). Recently, the concept that genomic instability due to a DNA repair defect caused by the genetic removal of *ERCC1* was involved in the development of vascular aging and age-related cardiovascular and cerebrovascular diseases was confirmed in the work of Bautista-Niño et al. (7). Furthermore, mice with *ERCC1* deficiency also had elevated serum cholesterol level and change in the expression of genes involved in the metabolism of cholesterol, which were associated with vascular stiffness and have been considered traditional risk factors for ischemic stroke (8, 9). Consequently, based on the above findings, DNA excision repair-related gene variations may alter its mRNA and protein expression, which in turn accelerates vascular stiffness and increases ischemic stroke risk.

DNA excision repair includes base excision repair (BER) and NER. Ghosh et al. found lower expression of X-ray repair cross-complementing 1 (*XRCC1*) proteins required for BER in brain samples from human individuals who died of ischemic stroke compared with individuals who died of non-neurological causes. Furthermore, there was serious brain damage in *XRCC1*^+/−^ mice than in wild-type mice. These results indicated that impaired BER might be a risk factor for ischemic stroke. Furthermore, impaired *XRCC1* was associated with increased genetic susceptibility to ischemic stroke (10). NER is another important DNA repair pathway and is critical in repairing various DNA lesions. *ERCC1*, a key component as a highly conserved rate-limiting enzyme in NER, forms a heterodimer with excision repair cross-complimentary group 4 (*ERCC4*) and functions as a structure-specific endonuclease in the incision step of the DNA repair system (11). He et al. established an *ERCC1* gene knockdown and overexpression rat middle cerebral artery occlusion (MCAO) model and found both endogenous and exogenous *ERCC1* could protect the brain against ischemic injury. There was the first evidence that *ERCC1* has a protective role in the pathophysiological process of ischemic stroke in the MCAO rat (12). In our previous study, we found that the *ERCC4* expression levels were significantly lower in patients with ischemic stroke than in healthy controls, and the 30028T/C polymorphism *(rs1799801*) of ERCC4 might be associated with ischemic stroke susceptibility. It was suggested that ERCC4, as an important component of the *ERCC1/ERCC4* heterodimer and a rate-limiting enzyme in the NER pathway, might play a key role in the pathophysiological process of ischemic stroke and that its variations were likely to increase ischemic stroke risk (13). Therefore, we hypothesized that ERCC1 polymorphism and its expressions may also be associated with ischemic stroke risk.

Genome-wide association studies have been conducted to confirm the relationship between genetic polymorphisms and susceptibility to ischemic stroke (14, 15). Recently, several common and putatively functional single nucleotide polymorphisms (SNPs) of *ERCC1* have been identified, of which *ERCC1* C118T (*rs11615*) at exon 4 without amino acid change and *ERCC1* C8092A (rs3212986) located at the 3'-untranslated region (3'-UTR) were likely to have some effects on *ERCC1* mRNA expression, which played an important role in cancer susceptibility, clinical phenotype diversity, and therapy (16–18). As everyone knows, the 3'-UTR of a gene is strongly related to regulating transcription and translation. The importance of the polymorphism from 3'-UTR is not only from their direct modification of the related gene functions but also from their genetic linkage with other causative germ-line mutations (19). The previous study showed that the *rs3212986* polymorphism of *ERCC1* has been associated with better objective response to chemotherapy among Asian patients with cancer, while studies on Caucasians have not found a significant association (20). Chen et al. conducted the first study to report a significant association of the *rs3212986* polymorphism in *ERCC1* with the risk of brain tumors and considered that the A/C *rs3212986* polymorphism, which may affect mRNA stability for *ERCC1*, also results in an amino acid substitution of lysine to glutamine in nucleolar protein (*ASE-1*) and T-cell receptor complex subunit CD3 epsilon-associated signal transducer (*CAST*) (21). It was suggested that the *rs3212986* polymorphism of *ERCC1* might play an important role in disease susceptibility. Nevertheless, there was little information about the association of ischemic stroke susceptibility with the *rs3212986* polymorphism located in the 3'-UTR of *ERCC1* and strongly related to altering its mRNA and protein expression. In the current study, we conducted a case-control study to detect *ERCC1* mRNA and protein levels and evaluated associations between the *ERCC1 rs3212986* polymorphism and ischemic stroke susceptibility in the Chinese Han population.

## 2. Materials and methods

### 2.1. Study population

The peripheral blood samples were collected from 513 patients with ischemic stroke and 550 healthy controls in the Affiliated Hospital of North Sichuan Medical College. All fresh peripheral blood samples were collected in an EDTA-coated vacutainer tube. The fresh peripheral blood samples of patients with ischemic stroke were collected within 24 h after the stroke. A total of 84 patients with ischemic stroke and 84 healthy controls were randomly selected to analyze *ERCC1* mRNA and protein levels.

All patients with ischemic stroke were diagnosed and confirmed by computed tomography and/or magnetic resonance according to ICD-9-CM codes 433, 434, and 436 (in accordance with ICD-10 codes I63.0-9). Ischemic stroke subtypes, including large artery atherosclerosis (LAA), cardioembolism (CE), small artery occlusion (SAA), stroke of other determined etiology (SOE), and stroke of undetermined etiology (SUE), were classified based on the criteria of Trial of Org 10172 in acute stroke treatment. Ischemic stroke patients with transient ischemic attack, hemorrhagic stroke, and other strokes caused by tumors, blood disease, traumatic brain injuries, and cerebrovascular malformations were excluded. Healthy controls were free from ischemic stroke, clear ischemic changes, stroke symptoms, malignant tumor, severe hepatic and renal dysfunction, and immunological disease. All individuals enrolled were from the Han Chinese ethnic group. Informed consent was obtained from all participants, and the study protocol and consent form were approved by the Ethics Committee of North Sichuan Medical College.

### 2.2. Clinical data collection

Clinical data of patients with ischemic stroke and healthy controls were collected, including demographic information (age, sex, height, weight, living area, ethnicity, former/current smoking or drinking, etc.), medical history (hypertension, diabetes, coronary heart disease, prior ischemic stroke, hyperlipidemia, etc.), the main laboratory data of white blood cells (WBC), neutrophil percentage, platelet (PLT), total cholesterol (TC), triglyceride (TG), low-density lipoprotein cholesterol (LDLC), high-density lipoprotein cholesterol (HDLC), very low-density lipoprotein (VLDL), red blood cell (RBC), and hemoglobin (HGB). Body mass index (BMI) was calculated as baseline weight in kilograms divided by squared height in meters (kg/m^2^).

### 2.3. Quantitative real-time polymerase chain reaction

Peripheral blood mononuclear cells (PBMCs) were isolated from the peripheral venous blood of enrolled individuals by Ficoll-Hypaque density gradient centrifugation with lymphocyte separation medium (TBD, China). Total RNA was extracted from PBMCs using RNAiso Plus (Takara, Japan) and subsequently reverse-transcribed using PrimeScript™ RT reagent Kit with gDNA Eraser (Takara, Japan) according to the manufacturer's instructions.

qPCR was performed using the SYBR^®^ Premix Ex Taq™ II kit (Takara, Japan) in the LightCycler^®^96 PCR Machine (Roche, Germany) according to the manufacturer's instructions. β-actin was served as an internal standard. The primer sequences of *ERCC1* were F: 5'-GAGCCTCAAGGGAAAGACTGC-3' and R: 5'-TCGCCCTGCTCTATGCTCTACT-3' (size: 132 bp). The primer sequences of β*-actin* were F: 5'-CCACGAAACTACCTTCAACTCC-3' and R: 5'-GTGATCTCCTTCTGCATCCTGT-3' (size: 132 bp). The PCR amplification was performed with a total volume of 20 μl, containing 0.4 μl each primer (0.4μM), 2μl template cDNA, 10 μl SYBR Premix Ex Taq (X2), and 0.4 μl ROX Reference Dye, adding ddH_2_O to 20 μl total reaction volume. The amplification conditions were as follows: 1 cycle of predenaturation at 95°C for 60 s, 40 cycles of denaturation at 95°C for 10 s, annealing at 60°C for 10 s, and a final extension at 72°C for 10 s. The standard curves of *ERCC1* and β*-actin* were generated by detecting geometric serial dilutions in the range of 10^6^-10^1^. The efficiencies of amplification were calculated according to the formula E=[10^(−1/*S*)^]^−1^, with S being the slope of the standard curve. A melting curve analysis was performed at 95°C for 5 s, 65°C for 30 s, and 97°C for 1 s. Amplification size was 132 bp for both *ERCC1* and β-actin. No template samples were used as blank or negative controls. All reactions were performed three times, and the average value of each sample was used for further data analysis. The *ERCC1* mRNA relative expression levels were calculated using the 2^−Δ*ΔCT*^ method (ΔCT=CT^*ERCC*1^–CT^β−*actin*^; ΔΔCT = ΔCT^case^-ΔCT^control^).

### 2.4. Enzyme-linked immunosorbent assay

Peripheral venous blood was centrifuged at 1,500 rpm for 10 min at 4°C to separate plasma. *ERCC1* protein levels in plasma were measured using the ELISA kits (USCN, China) according to the manufacturer's instructions at 450 nm in the microplate reader (Bio-Rad, USA). The sensitivity of the *ERCC1* ELISA kit was 0.056 ng/ml. The cut-off value was set as the mean absorbance in the negative controls. Concentrations were calculated from standard curves. *ERCC1* protein level was calculated using the following equation: *ERCC1* = 1.154^*^OD^2^ + 0.5232^*^OD + 0.1886 (*R*^2^ = 0.9984). OD was represented by optical density. The results were expressed in nanograms per milliliter. Each sample was repeated three times.

### 2.5. Genotyping

Genomic DNA was extracted from 200 μl of peripheral venous blood using a commercial DNA isolation kit (BioTeke, China) according to the manufacturer's instructions and then stored at −20°C.

PCR restriction fragment length polymorphism (*PCR-RFLP*) was used to detect the *ERCC1 rs3212986* genotype. The PCR primers were F: 5′-ACCCCACTCTAGATTTACCCAGGAA-3′ and R: 5′-AAGAAGCAGAGTCAGGAAAGC-3′ (size: 442 bp). PCR was performed in a total reaction volume of 10 μl, containing 100 ng genomic DNA, 0.2 μl each primer (0.4 μM), and 4 μl 2 × Taq PCR MasterMix (Qiagen, Germany), adding ddH_2_O to 10 μl total reaction volume. After an initial denaturation at 94°C for 3 min, the DNA was amplified for 40 cycles at 94°C for 30 s, 65°C for 30 s, and 72°C for 60 s, followed by a final extension at 72°C for 5 min in BIO-RAD PCR amplification instrument (BIO-RAD, USA).

The PCR products were digested with 2U *MboII* (New England Biolabs, USA) at 37°C in a reaction volume of 10 μl for 4 h. The restriction enzyme *MboII* was used to identify the genotype of *rs3212986*, and the 442 bp, 315 bp, and 127 bp restriction fragments were obtained. In the presence of the A allele, the PCR products were divided into two fragments of 315 bp and 127 bp, while products containing the C allele were not cleavable and remained a 442 bp fragment. The variants (sizes of bands for each genotype) were determined using 3% agarose gel electrophoresis. Allele and genotype frequencies of *ERCC1 rs3212986* were determined by direct counting. For quality control and validation purposes, more than 10% of PCR-amplified samples were confirmed by DNA sequencing analysis, with 100% reported reproducibility (Supplementary Figure S1).

### 2.6. Statistical analysis

All data were presented as the mean±standard deviation (SD) for continuous variables and as numbers or percentages (%) for categorical variables. Differences between the case and control groups were evaluated by Student's *t*-test or the rank-sum test for continuous variables and the chi-square test or Fisher's exact test for categorical variables. The Hardy–Weinberg equilibrium (HWE) was detected by the chi-square test. Associations between the *ERCC1 rs3212986* polymorphism and ischemic stroke susceptibility were analyzed using odds ratios (ORs) and 95% confidence intervals (CIs) calculations for the allele contrast (A vs. C), dominant (AA+CA vs. CC), recessive (AA vs. CC+CA), and additive (AA+CC vs. CA) genetic models. SPSS version 17.0 (SPSS, Inc., Chicago, IL) was used for statistical analysis, and a two-sided *P-*value of < 0.05 was considered statistically significant.

## 3. Results

### 3.1. Clinical characteristics of the study population

The clinical characteristics of the study population were summarized in Table 1. The mean age was 68.23 ± 10.16 years for the patients with ischemic stroke (289 male patients and 224 female patients) and 67.23 ± 7.56 years for the control subjects (289 male patients and 261 female patients). There was no significant difference in age and gender between the patients with ischemic stroke and the control subjects (*P* > 0.05).

**Table 1:** Characteristics of the study population.

**Characteristics**	**Ischemic stroke patients (*N =* 513)**	**Controls (*N =* 550)**	***P*-value**
**Demographics**			
Male, *N* (%)	289(56.34%)	289(52.55%)	0.218
Age, mean (SD), years	68.23 ± 10.16	67.23 ± 7.56	0.07
**Past medical history**, ***N*** **(%)**			
Prior ischemic stroke	115(22.42%)		
Hypertension	328(63.94%)	179(32.55%)	< 0.000
Coronary heart disease	54(10.53%)	29(5.27%)	0.002
Hyperlipidemia	117(22.81%)	60(10.91%)	< 0.000
Diabetes	87(15.59%)	50(9.09%)	< 0.000
BMI>30	93(18.13%)	46(8.36%)	< 0.000
Former/current smoking	86(16.76%)	92(16.73%)	1.000
Former/current drinking	190(37.04%)	106(19.27%)	< 0.000
**Main laboratory data, mean (SD)**			
WBC, 10^9^ /L	7.29 ± 2.64	5.67 ± 1.52	< 0.000
Neutrophil percentage, %	66.53 ± 13.06	61.41 ± 8.80	< 0.000
PLT, 10^12^ /L	155.71 ± 56.71	169.06 ± 51.41	< 0.000
TG, mmol/L	1.60 ± 0.94	1.24 ± 0.78	< 0.000
TC, mmol/L	4.43 ± 1.02	4.46 ± 0.67	0.576
HDLC, mmol/L	1.13 ± 0.37	1.33 ± 0.45	< 0.000
LDLC, mmol/L	2.60 ± 0.95	2.63 ± 0.60	0.494
VLDL, mmol/L	0.79 ± 0.59	0.62 ± 0.46	< 0.000
RBC, 10^12^/L	4.35 ± 0.58	4.90 ± 2.70	< 0.000
HGB, g/L	127.93 ± 24.59	141.06 ± 17.62	< 0.000
**TOAST classification**, ***N*** **(%)**			
LAA	97(18.91%)		
SAA	300(58.48%)		
CE	53(10.33%)		
SOE	29(5.65%)		
SUE	34(6.63%)		

SD, standard deviation; BMI, body mass index; WBC, white blood cells; PLT, platelet; TC, total cholesterol; TG, triglyceride; LDLC, low density lipoprotein cholesterol; HDLC, high density lipoprotein cholesterol; VLDL, very low density lipoproteinet; RBC, red blood cell; HGB, hemoglobin; TOAST, Trial of Org 10172 in Acute Stroke Treatment; LAA, large artery atherosclerosis; CE, cardioembolism; SAA, small artery occlusion; SOE, stroke of other determined etiology; SUE, stroke of undetermined etiology.

Hypertension, coronary heart disease, hyperlipidemia, diabetes, BMI> 30, and former/current drinking were more commonly seen in the patients with ischemic stroke than in the controls (*P* < 0.05). The levels of WBC, neutrophil percentage, TG, and VLDL in patients with ischemic stroke were higher than those in the control group (*P* < 0.05). Nevertheless, the levels of PLT, HDLC, RBC, and HGB in patients with ischemic stroke were lower than in the control group (*P* < 0.05). The characteristics of the study population for analyzing *ERCC1* expression levels are seen in Supplementary Table S1.

### 3.2. *ERCC1* mRNA and protein expression levels

The melting curves of *ERCC1* and ß*-actin* showed a single peak, and the melting temperatures (Tm) were 85.9 and 82.0°C, respectively. Additionally, the slopes of the standard curve of *ERCC1* and ß*-actin* were −3.331 and −3.337, respectively. The amplification efficiencies of *ERCC1* and ß*-actin* were 0.995 and 0.994, respectively, according to the conversion formula. Consequently, the amplification specificity and efficiency complied with the requirements of the qPCR assay.

**Figure 1:**
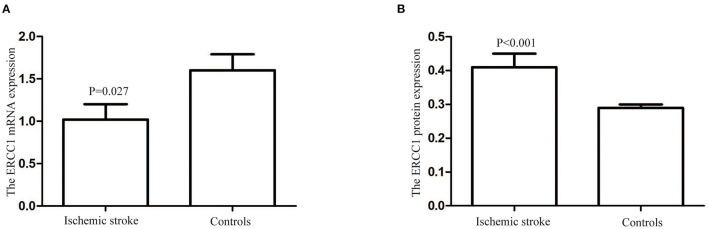
The *ERCC1* mRNA and protein levels in patients with ischemic stroke and controls. **(A)** The *ERCC1* mRNA relative expression level in patients with ischemic stroke was lower than in the controls (*P* = 0.027). **(B)** The *ERCC1* protein levels in patients with ischemic stroke were higher than in the controls (*P* < 0.001).

The *ERCC1* mRNA relative expression levels in patients with ischemic stroke and controls were 1.02 ± 0.18 and 1.60 ± 0.19, respectively. The *ERCC1* mRNA's relative expression level in patients with ischemic stroke was lower than that in the control group (*P* = 0.027, Figure 1A). However, the level of *ERCC1* protein in ischemic stroke patients (0.41 ± 0.04 ng/mL) was higher than that in controls (0.29 ± 0.01 ng/ml) (*P* < 0.001, Figure 1B).

### 3.3. *ERCC1 rs3212986* polymorphism and ischemic stroke susceptibility

A total of 513 patients with ischemic stroke and 550 healthy controls were successfully genotyped. The allele and genotype distributions of the *ERCC1 rs3212986* polymorphism were summarized in Table 2. The distribution of genotype *rs3212986* was not in accordance with the Hardy–Weinberg equilibrium (HWE) in the control group (*P* < 0.05).

**Table 2:** Association between *ERCC1 rs3212986* polymorphism and ischemic stroke susceptibility.

	**Allele/genotype**	**Ischemic stroke patients (*N =* 513)**	**Controls (*N =* 550)**	**Crude OR (95 % CI)**	**Crude *P***	**Adjust OR (95 % CI)^a^**	**Adjust *P^*a*^***
	Allele				0.006		
	A	384	349	1.287(1.076–1.540)			
	C	642	751	1 (Ref)			
Genetic model	Genotype						
Recessive model	AA	80	36	2.638(1.744–3.989)	< 0.001	2.015(1.087–3.704)	0.026
	CC+CA	433	514	1 (Ref)		1 (Ref)	
Dominant model	AA+CA	304	313	1.101(0.863–1.406)	0.456	1.124(0.769–1.639)	0.554
	CC	209	237	1 (Ref)		1 (Ref)	
Additive model	AA+CC	289	273	1.309(1.028–1.667)	0.031	1.494(0.982-2.273)	0.061
	CA	224	277	1 (Ref)		1 (Ref)	

Ref, reference; OR, odds ratio; CI, confidence interval. ^a^Adjusted for demographics information (age and gender), past medical history (hypertension, coronary heart disease, hyperlipidemia and diabetes), laboratory data (WBC, neutrophil percentage, TG, VLDL, PLT, HDLC, RBC and HGB), BMI>30 and former/current drinking.

The A allele of *rs3212986* significantly was associated with an increased risk of ischemic stroke (OR = 1.287, 95% CI = 1.076–1.540, *P* = 0.006). The association between the *ERCC1 rs3212986* polymorphism and ischemic stroke susceptibility was found in both recessive (OR = 2.638, 95% CI = 1.744–3.989, *P* < 0.001) and additive (OR = 1.309, 95% CI = 1.028–1.667, *P* = 0.031) models, respectively.

After adjusting for demographic information (age and gender), medical history (hypertension, coronary heart disease, hyperlipidemia, and diabetes), main laboratory data (WBC, neutrophil percentage, TG, VLDL, PLT, HDLC, RBC, and HGB), and other variables (BMI>30 and former/current drinking), the significant associations between *ERCC1 rs3212986* polymorphism and ischemic stroke susceptibility were also obtained in the recessive model (OR = 2.015, 95% CI = 1.087–3.704, *P* = 0.026), as shown in Table 2.

### 3.4. *ERCC1 rs3212986* polymorphism and its expression

The above data showed that the *ERCC1 rs3212986* polymorphism was associated with ischemic stroke susceptibility. However, the effects of the *rs3212986* polymorphism on gene expression were unclear.

**Figure 2:**
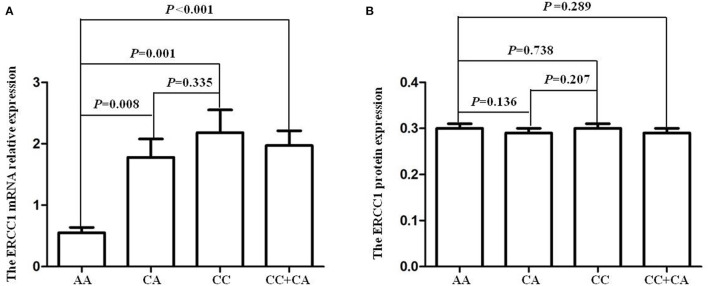
*ERCC1 rs3212986* polymorphism and its expression levels. **(A)** The individuals carrying with C allele (CC/CA genotypes) of *ERCC1 rs3212986* polymorphism showed higher mRNA expression levels than those carrying the AA genotype (CC vs. AA, *P* = 0.001; CA vs. AA, *P* = 0.008; CC/CA vs. AA, *P* < 0.001). **(B)** None of the genotypes affected *ERCC1* protein expression levels (*P* > 0.05).

The results of this study showed that the *ERCC1* mRNA levels in individuals with AA, CA, CC, and CC/CA genotypes were 0.55 ± 0.09, 1.78 ± 0.30, 2.18 ± 0.37, and 1.97 ± 0.24, respectively. The *ERCC1* mRNA levels in individuals carrying the C allele (CC/CA genotypes) of the *rs3212986* polymorphism were higher than in those with the AA genotype (*P* < 0.05, Figure 2A). However, there was no significant difference in *ERCC1* plasma protein levels among different genotypes of *ERCC1 rs3212986* (*P* > 0.05, Figure 2B).

## 4. Discussion

This study indicated that the A allele of *ERCC1 rs3212986* was associated with increased ischemic stroke risk, and the altered *ERCC1* mRNA expression level caused by the *ERCC1 rs3212986* polymorphism might participate in the pathophysiological process of ischemic stroke.

There was the first evidence that A allele of the *ERCC1 rs3212986* polymorphism was associated with increased ischemic stroke risk. Similar results were observed after adjusting for clinical data, including demographic information, medical history, and main laboratory data. It was suggested that A allele of the *ERCC1 rs3212986* polymorphism might be a risk factor for ischemic stroke. The *ERCC1 rs3212986* polymorphism, located on the 3'-UTR of the *ERCC1* gene, has been widely studied in previous studies (16, 22, 23) and might be involved in the potential pathophysiological mechanism in ischemic stroke. *ERCC1 rs3212986* polymorphism might affect the DNA repair capacity and interfere with the NER pathway by regulating *ERCC1* transcription and translation and/or altering *ERCC1* biological activity (24, 25). The *rs3212986* polymorphism associated with altered *ERCC1* mRNA expression was found in different tissues through the GTEx database, such as skin, arteries, fibroblasts, testis, and adipose subcutaneous tissue. The site of *rs3212986* in the column “QTLhits” indicated that the site may be the expression quantitative trait loci (eQTLs) of the *ERCC1* gene in the HaploReg database, which may regulate the expression of *ERCC1*. In addition, Yu et al. predicted the *ERCC1* mRNA secondary structure between two genotypes of *rs3212986* by bioinformatics software and found that *ERCC1 rs3212986* genetic variation may affect DNA repair capacity by altering the folded stem-loop structure consisting of six repeats of “GCT”, which could impact the 18-base sequence in the 3′-UTR of *ERCC1* (25). Furthermore, *ERCC1* mRNA expression may be regulated by miRNAs, which could also be affected by the *rs3212986* polymorphism in the target complementary sequence (16, 26). There were some candidate miRNAs in the *ERCC1* 3′-UTR region identified by online websites (miRNASNP-v3, miRBase, and TargetScan Release 7.2), including 4 miRNAs of target gain (hsa-miR-3185, hsa-miR-7-5p, hsa-miR-10522-5p, and hsa-miR-6077) and 2 miRNAs of target loss (hsa-miR-6828-5p and hsa-miR-671-5p) in 3'-UTR of *ERCC1*. The binding of *rs3212986* with 4 miRNAs of target gain in the 3'-UTR of *ERCC1* might result in lower *ERCC1* mRNA expression with the AA genotype. Therefore, the *ERCC1 rs3212986* polymorphism may alter its mRNA and protein expression and promote endothelial cell dysfunction, vascular senescence, and plaque rupture, which eventually contribute to accelerated vascular stiffness and increased ischemic stroke risk.

In the current study, a lower relative expression level of *ERCC1* mRNA in patients with ischemic stroke was observed. Unfortunately, a similar alteration of the expression level of the *ERCC1* protein failed to be identified. To investigate whether the *ERCC1 rs3212986* polymorphism alters *ERCC1* gene expression, the mRNA and protein expression levels between different genotypes were analyzed in this study. We found that *ERCC1* mRNA levels were higher in individuals with the C allele of the *ERCC1 rs3212986* polymorphism (CC/CA genotype) than in individuals with the AA genotype. The findings confirmed the mechanism of *ERCC1 rs3212986* polymorphism in regulating *ERCC1* transcription. In addition, genetic mutations might cause the activity of some proteins or enzymes to decrease or even disappear (27). The previous *in vitro* studies examined the structural stability of proteins by molecular dynamics (MD) to understand the structure-function relationship and found that some non-synonymous single nucleotide polymorphisms limited the activity of proteins (28). However, no significant differences in *ERCC1* protein levels were found among subjects carrying different *ERCC1 rs3212986* genotypes. The translation or biological activity of *ERCC1* protein in different *ERCC1 rs3212986* genotypes needs to be confirmed *in vitro* studies in the future.

The discordance between the transcriptome and the proteome was also observed in a previous study and was considered to be strongly affected by the lack of temporal synchronization between the transcriptional and translational regulation levels (29, 30). In mammals, the correlation between expression levels of mRNA and protein was relatively weak, with a correlation coefficient of ~0.40, which indicated that ~40% of the variation in protein levels can be explained by mRNA abundances (31, 32). Therefore, transcription and translation were far from having a linear and simple relationship. The complicated and changeable mechanisms of transcription and translation generated a big regulator control system of gene expression that enhanced or repressed the synthesis of proteins from a certain copy number of mRNA molecules. Some proteins maximized all these processes with high and stable translation rates, but the transcription rates were relatively low and opposed mRNA stability. Initial anchoring of the ribosome onto the mRNA depended on the complementary binding of the Shine-Dalgarno (SD) sequence, which was considered an important factor to impact the efficiency of protein biosynthesis that contributed to 1.9–3.8% of the total variation of the mRNA-protein correlation (31, 33). Many details of the complicated biological processes of transcription and translation still need further investigation. Additionally, transient transcription of genes was limited, especially when genetic dysfunction is encountered (34). The transcription might be weak, even would not be induced or activated due to *ERCC1* deficiency, if there were severe ischemia, hypoxia, and stress stimulation in patients with ischemic stroke. Finally, the body failed to transcribe plenty of *ERCC1* mRNAs to provide the template for translation. Certainly, we cannot ignore that the sample heterogeneity may be the cause of the expression difference between mRNA in peripheral blood mononuclear cells and protein in plasma. Importantly, *ERCC1* expression levels in peripheral blood only relatively reflect the repair situation of patients with ischemic stroke to a certain extent, and future studies are needed to detect *ERCC1* expression in atherosclerotic plaque or edema area, ischemic penumbra, or ischemic core in ischemic stroke animal models and clarify the detailed function of the *ERCC1* gene in the pathophysiology of ischemic stroke.

In addition, the results indicated that neutrophils, as important laboratory indexes for inflammation, were integral to the pathology in patients with ischemic stroke, which was consistent with previous studies (35). In murine models of ischemic stroke, neutrophils were the primary cells recruited to the core area and penumbra area at the onset of ischemic stroke, accompanied by local production of cytokines or chemokines (36). Local inflammatory immune responses initiated in brain tissue might further exacerbate tissue damage and disrupt the brain-blood barrier (BBB) (37). Neutrophils might play a key role in the induction or promotion of inflammation-induced tissue damage in the presence of inappropriate and/or overactivation (38, 39).

However, there were some limitations in the study, which must be taken into account when interpreting the results. First, the results were only from a single hospital population of Han nationality, which might result in selection bias and limit the applicability to other ethnic groups. Second, some important clinical data, such as lifestyle, working conditions, or economic pressure, and some other ischemic stroke-related risk factors were lacking due to a retrospective study, which limited our evaluation of gene-environment interactions. Third, the plasma and PBMC samples from patients were selected to evaluate the biological function of the polymorphism rather than cell lines, which might influence experimental results. Fourth, the distribution of genotype *rs3212986* was not in accordance with the Hardy-Weinberg equilibrium (HWE) in the control group. As everyone knows, insufficient samples, genotyping error, and sample selection bias are the main factors of an unbalanced distribution of HWE. However, insufficient samples and genotyping errors were excluded by the GAS Power Calculator and by rechecking genotypes, respectively. Therefore, we carefully analyzed the clinical characteristics of the control group. We found that older, healthy people were included in the control group to match the age of patients with ischemic stroke. The sample selection bias was likely to be the source of the unbalanced distribution of genotype *rs3212986* in the control group. Nevertheless, the significant associations between *ERCC1 rs3212986* polymorphism and ischemic stroke susceptibility still remained after adjusting for the age factor. Furthermore, the results need to be verified by multicenter, randomized, and large-sample prospective studies.

## 5. Conclusion

The findings indicated that *ERCC1 rs3212986* polymorphism was associated with ischemic stroke susceptibility in a Chinese Han population, and A allele of *rs3212986* might be related to increasing ischemic stroke risk. The altered *ERCC1* mRNA expression level caused by the *ERCC1 rs3212986* polymorphism might participate in the pathophysiological process of ischemic stroke. Further prospective studies with a larger sample from multiple centers might enhance our results.

## Data availability statement

The original contributions presented in the study are included in the article/Supplementary material, further inquiries can be directed to the corresponding authors.

## Ethics statement

The studies involving human participants were reviewed and approved by Ethics Committee of North Sichuan Medical College. The patients/participants provided their written informed consent to participate in this study. Written informed consent was obtained from the individual(s) for the publication of any potentially identifiable images or data included in this article.

## Author contributions

X-DD, YL, and YM contributed to the conception and design of the study. J-LK, T-YC, QG, Z-LZ, M-LX, and L-ZW collected samples and performed the related experimental operations. X-DD, WZ, and HL performed the statistical analysis. X-DD and J-LK wrote the first draft of the manuscript. X-DD was mainly responsible for manuscript revision. All authors read and approved the submitted version.

## Supplementary material

Supplementary Figure S1 The sequence maps of *ERCC1 rs3212986* polymorphism. **(A)** The sequence map of CC genotype. **(B)** The sequence map of CA genotype. **(C)** The sequence map of AA genotype.

Supplementary Table S1 Characteristics of the study population for analyzing ERCC1 expression levels.
