# Supplementary figures and images for: ERCC1 polymorphism and its expression associated with ischemic stroke in Chinese population
Source: Front Neurol. 2023 Jan 12;13:998428. doi: 10.3389/fneur.2022.998428 (PMC9878395; doi:10.3389/fneur.2022.998428)

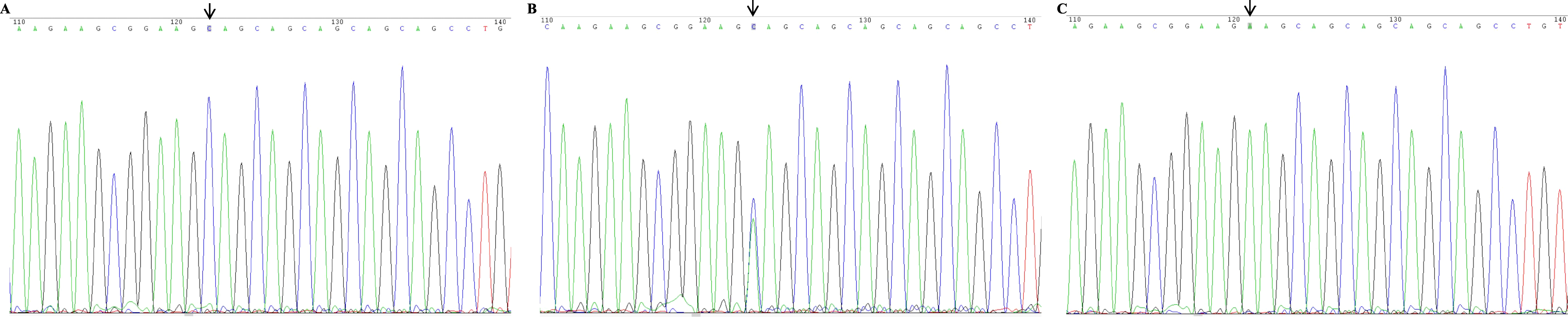

Supplement: Supplementary Figure S1 — The sequence maps of ERCC1 rs3212986 polymorphism. (A) The sequence map of CC genotype. (B) The sequence map of CA genotype. (C) The sequence map of AA genotype. [file Image_1.TIF]
