# Supplementary material for: ERCC1 polymorphism and its expression associated with ischemic stroke in Chinese population
Source: Front Neurol. 2023 Jan 12;13:998428. doi: 10.3389/fneur.2022.998428 (PMC9878395; doi:10.3389/fneur.2022.998428)
Supplement: Supplementary Table S1 — Characteristics of the study population for analyzing ERCC1 expression levels. [file Table_1.docx]

**TABLE S1** Characteristics of the study population for analyzing *ERCC1* expression levels

| **Characteristics** | **Ischemic stroke patients(N=84)** | **Controls(N=84)** | ***P* value** |
| --- | --- | --- | --- |
| Demographics | | |  |
| Male, N(%) | 50（59.52%） | 51（60.71%） | 1.000 |
| Age, mean(SD), years | 70.64±7.28 | 68.0±7.58 | 0.835 |
| Past medical history, N(%) |  |  |  |
| Prior ischemic stroke | 31（36.9%） |  |  |
| Hypertension | 70（83.3%） | 46（54.76%） | <0.000 |
| Coronary heart disease | 5（5.95%） | 3（3.57%） | 0.72 |
| Hyperlipidemia | 15（17.85%） | 7（8.33%） | 0.108 |
| Diabetes | 23（27.38%） | 14（14.67%） | 0.136 |
| BMI>30 | 18（21.43%） | 10（11.9%） | 0.146 |
| Former/current smoking | 15（17.86%） | 13（15.48%） | 0.836 |
| Former/current drinking | 33（39.29%） | 18（21.43%） | 0.018 |
| Main laboratory data, mean(SD) |  |  |  |
| WBC, 10^9^ /L | 7.25±2.84 | 5.49±1.57 | <0.000 |
| Neutrophil percentage, % | 65.98±12.25 | 62.66±7.02 | 0.087 |
| PLT, 10^12^ /L | 149.73±51.02 | 165.79±51.62 | 0.085 |
| TG , mmol/L | 2.13±0.98 | 1.17±0.59 | <0.000 |
| TC, mmol/L | 4.61±1.20 | 4.36±0.57 | 0.166 |
| HDLC , mmol/L | 1.03±0.30 | 1.32±0.33 | <0.000 |
| LDLC, mmol/L | 2.58±0.98 | 2.62±0.48 | 0.829 |
| VLDL, mmol/L | 0.90±0.59 | 0.66±0.61 | 0.028 |
| RBC, 10^12^ /L | 4.36±0.50 | 4.69±1.57 | 0.079 |
| HGB, g/L | 135.65±18.37 | 142.35±16.18 | 0.037 |
| *Note:* SD, standard deviation; BMI, body mass index; WBC, white blood cells; PLT, platelet; TC, total cholesterol; TG, triglyceride; LDLC, low density lipoprotein cholesterol; HDLC, high density lipoprotein cholesterol; VLDL, very low density lipoproteinet; RBC, red blood cell; HGB, hemoglobin. | | | |
